# Quantification of hepatic fat: evaluation of different magnetic resonance imaging measurement strategies in cases of homogeneous and heterogeneous distribution

**DOI:** 10.1590/0100-3984.2024.0009-en

**Published:** 2024-11-18

**Authors:** Eloa de Castro Noguerol, Luis Ronan Marquez Ferreira de Souza, Valdair Francisco Muglia, Jorge Elias Jr.

**Affiliations:** 1 Faculdade de Medicina de Ribeirão Preto da Universidade de São Paulo (FMRP-USP), Ribeirão Preto, SP, Brazil; 2 Universidade Federal do Triângulo Mineiro (UFTM), Uberaba, MG, Brazil

**Keywords:** Fatty liver, Non-alcoholic fatty liver disease, Magnetic resonance imaging, Esteatose hepática, Doença hepática gordurosa não alcoólica, Ressonância magnética

## Abstract

**Objective:**

To evaluate three different measurements strategies to quantify hepatic steatosis and to investigate the differences between homogeneous and heterogeneous forms of hepatic steatosis.

**Materials and Methods:**

Retrospective study conducted by magnetic resonance imaging review. We evaluated three different strategies measures for quantification of hepatic steatosis in two matched groups: homogeneous and heterogeneous steatosis. We considered *p* < 0.05 significance level in all made tests.

**Results:**

In heterogeneous steatosis group, the strategy with a region of interest (ROI) of 1 cm^2^ to measure the signal intensity in the most altered area showed significant variations in the quantification, while the average of four ROIs of 1 cm^2^ or representative target area in axial section did not vary significant. In diffuse hepatic steatosis, any strategy used showed no significant difference. The intraclass correlation coefficient ranged between 0.96 and 0.99, with 95% confidence interval of 0.93-0.99.

**Conclusion:**

The quantification of fat liver by magnetic resonance imaging using only one ROI is less representative, especially in heterogeneous steatosis. There was no significant difference between the average of four ROIs strategy and the strategy of representative segmentation area of parenchyma.

## Abstract (pt)

**Objetivo:**

Avaliar três estratégias diferentes de medidas para quantificação da esteatose hepática e verificar se existem diferenças entre as formas homogênea e heterogênea de esteatose.

**Materiais e Métodos:**

Estudo retrospectivo, realizado com base em revisão de exames de ressonância magnética. Foram avaliadas três diferentes estratégias de medidas para quantificação da esteatose hepática em dois grupos pareados: esteatose homogênea e esteatose heterogênea. Considerou-se nível de significância de *p* < 0,05 em todos os testes realizados.

**Resultados:**

No grupo de esteatose heterogênea, o uso de região de interesse (ROI) de 1 cm^2^ para medir a intensidade de sinal na área mais alterada apresentou variações significativas na quantificação, enquanto a média de quatro ROIs de 1 cm^2^ ou a segmentação de área representativa em corte axial não apresentaram variações significativas. Na esteatose hepática homogênea, qualquer estratégia utilizada não demonstrou diferença significativa. O coeficiente de correlação intraclasse variou entre 0,96 e 0,99, com intervalo de confiança 95% de 0,93-0,99.

**Conclusão:**

A quantificação da gordura hepática por ressonância magnética utilizando apenas uma ROI é menos representativa, principalmente na esteatose heterogênea. Não houve diferença significativa entre a obtenção da média de quatro ROIs e a segmentação de área representativa do parênquima.

## INTRODUCTION

Hepatic steatosis is the accumulation of triglycerides in hepatocytes, generally associated with alcoholic liver disease and with metabolic dysfunction-associated steatotic liver disease (MASLD), previously known as nonalcoholic fatty liver disease. Other, less common, conditions associated with hepatic steatosis include viral hepatitis, excessive use of certain medications, and genetic diseases^(1-5)^. The severity of the disease is related to the degree of fatty infiltration, and it can progress to steatohepatitis, cirrhosis, or hepatocellular carcinoma^(3-6)^.

In hepatic steatosis, the pattern of fatty infiltration can be homogeneous or heterogeneous. The homogeneous, or diffuse, presentation is the most common form and consists of uniform distribution of infiltration throughout the liver parenchyma^(7,8)^. The heterogeneous presentation may manifest as infiltration that is focal (geographic or nodular), multifocal, perilesional, subcapsular, intralesional, or perivascular, together with an area of focal preservation of the parenchyma amidst diffuse steatosis. In most cases of the heterogeneous form, the infiltration occurs in specific areas, such as near the falciform ligament, portal vein, or vesicular fossa. Although the heterogeneous pattern of distribution is not yet fully understood, it has been attributed to variations in hepatic venous circulation and can represent a diagnostic challenge, often making it difficult to differentiate it from tumors^(2,7-9)^.

Liver biopsy is still considered one of the reference standards for the diagnosis and assessment of the severity of hepatic steatosis, because it allows semiquantitative assessment of steatosis, as well as of the extent of inflammatory activity and fibrosis in the liver^(3,8,10)^. However, it is an invasive method with low representativeness, as well as considerable variation when more than one sample is analyzed from the same patient^(4,11,12)^. This variability can have a significant influence on the diagnosis, as well as on the staging of the disease, especially in patients with heterogeneous steatosis^(3,11-13)^.

Although it is possible to use computed tomography^(14)^ and ultrasound^(15)^ to quantify hepatic steatosis, magnetic resonance imaging (MRI) is considered a more accurate method that is well-established for detecting and quantifying liver fat, with chemical shift gradient-echo imaging being the most widely used technique^(1,12,16)^. This technique, known as the Dixon method, assesses the presence of liver fat by comparing the loss of signal intensity of the parenchyma in sequences known as in-phase and out-of-phase sequences. The amount of liver fat is determined by calculating the fat fraction, with the following formula:


FF=(in-phase SI-out-of-phase SI)∕2×in-phase SI

where *FF* is the fat fraction and *SI* is the signal intensity.

Despite the high specificity of the Dixon method, its sensitivity is limited in the presence of low fat levels and in patients with hepatic iron deposition, in whom the T2* effects will be significant^(13,17-20)^. More recent techniques, such as measurement of the fat fraction by proton density, are more accurate because of multiple corrections in the process of obtaining the signal, although they require additional financial investment because of the need to acquire a specific software package, limiting their wide-scale use^(20,21)^. One recently validated option for quantifying liver fat by MRI is measurement performed in two-dimensional gradient-echo sequences with MRQuantif software (https://imagemed.univ-rennes1.fr/en/mrquantif/download.php), the result showing a high correlation with the steatosis score and very close to the fat fraction estimated by histomorphometry^(21)^.

Several studies have demonstrated a good correlation between biopsy and the chemical shift technique in the detection and quantification of liver fat^(20-22)^. However, to our knowledge, there have been no studies demonstrating and characterizing the best way to obtain and measure signal intensity in order to calculate the fat fraction, especially when there is a heterogeneous pattern of fatty infiltration. The objective of the present study was to evaluate different measurement strategies for quantifying liver fat and to determine whether there is a difference between the strategy used for the assessment of homogeneous steatosis and that used for the assessment of heterogeneous steatosis.

## MATERIALS AND METHODS

This was a retrospective study, approved by the local research ethics committee. We selected abdominal MRI examinations that resulted in a diagnosis of hepatic steatosis, carried out between January 2012 and January 2014, available from the image bank of our facility. The presence of liver fat was verified using the chemical shift technique, and the diagnosis of hepatic steatosis was based on the presence of a fat fraction greater than or equal to 9%.

The examinations were performed in a high-field (1.5-T) MRI scanner (Achieva; Philips Medical Systems, Best, The Netherlands), with the following parameters: T1-weighted sequence in the axial plane, double-echo, in-phase (echo time = 4.6 ms) and out-of-phase (echo time = 2.3 ms), spoiled gradient echo (repetition time = 111 ms; flip angle = 80°; slice thickness = 6 mm; interslice gap = 7%; 30 slices for each echo, with a breath hold for 29 s).

All examinations were initially reviewed by a radiologist who was a specialist in abdominal imaging. Examinations with movement or magnetic susceptibility artifacts that hindered adequate evaluation were excluded from the analysis, as were those in which there were multiple liver lesions of another nature that could not be omitted from the signal intensity measurement area.

The examinations included in the study were separated into two groups, according to the pattern of fatty infiltration: homogeneous; and heterogeneous. With the exception of the diffuse pattern, all other forms of fatty infiltration were included in the heterogeneous group.

In both groups, we evaluated three different measurement strategies to obtain the fat fraction, all using in-phase and out-of-phase sequences to calculate the liver fat fraction, with the aformentioned formula.

Strategies 1 and 2, as described below, were applied at different times by two radiologists who were specialists in abdominal imaging, working independently. The third strategy was applied in a semi-automated manner with the software Display, by a third examiner, a radiology technician with an advanced degree, who analyzed the images from both groups.

**Figure 1:**
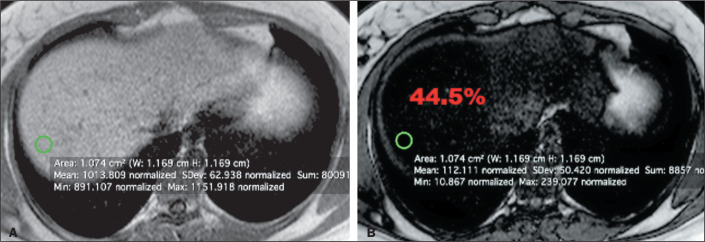
Strategy 1 in the homogeneous group. In-phase and out-of-phase axial images of the liver (A and B, respectively). Each green circle represents the selected 1 cm^2^ ROI with the values provided by MRI below. The calculated fat fraction value appears in red.

**Figure 2:**
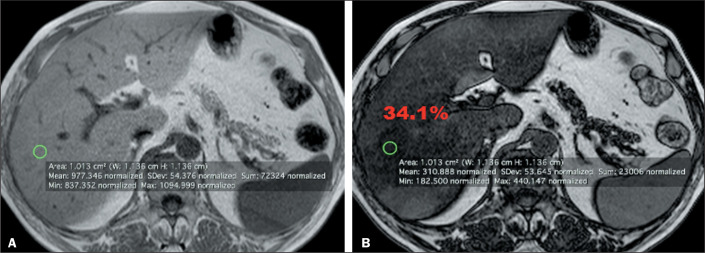
Strategy 1 in the heterogeneous group. In-phase and out-of-phase axial images of the liver (A and B, respectively). Each green circle represents the selected 1 cm^2^ ROI with the values provided by MRI below. The calculated fat fraction value appears in red.

**Strategy 1.** A region of interest (ROI) of 1 cm^2^ was selected at a determined point in the liver parenchyma. In the homogeneous group, the ROI was randomly selected from the parenchyma (Figure 1). In the heterogeneous group, the ROI was obtained at the point in the liver parenchyma identified by each observer as having the greatest fatty infiltration (Figure 2).

**Figure 3:**
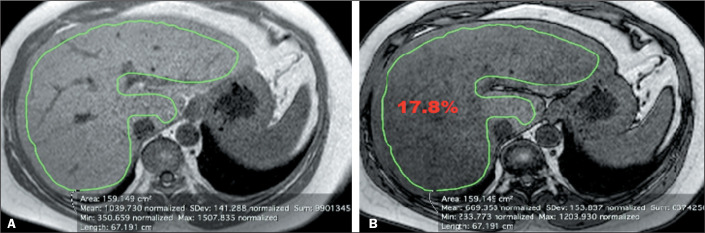
Strategy 2 in the homogeneous group. In-phase and out-of-phase axial images of the liver (A and B, respectively). The green area represents the manually selected ROI with the values provided by MRI below. The calculated fat fraction value appears in red.

**Figure 4:**
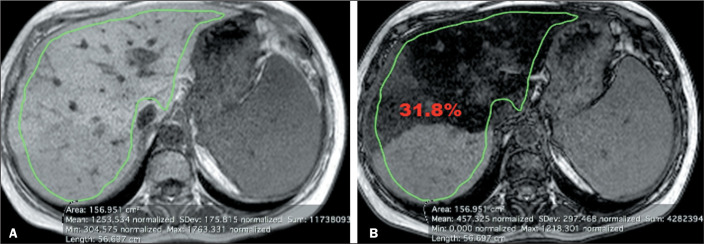
Strategy 2 in the heterogeneous group. In-phase and out-of-phase axial images of the liver (A and B, respectively). The green area represents the manually selected ROI with the values provided by MRI below. The calculated fat fraction value appears in red.

**Strategy 2.** An ROI was manually selected from axial MRI slices of the liver. In the homogeneous group, the ROI was selected in the section defined by the observer as the most central part of the liver, covering the largest possible amount of parenchyma within the area, in order to obtain the mean signal intensity for the entire ROI (Figure 3). In the heterogeneous group, the ROI was selected to encompass the entire liver parenchyma in a section defined by each observer as the point with the greatest heterogeneous fatty infiltration, in order to obtain the mean signal intensity for the entire ROI (Figure 4).

**Figure 5:**
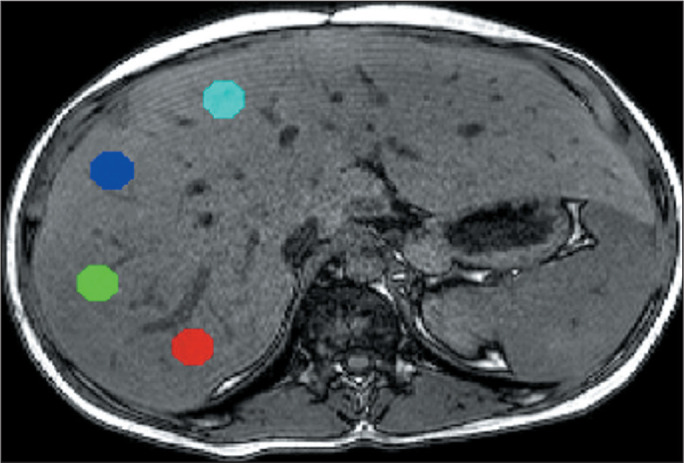
Strategy 3. Axial section of the liver with the four ROIs automatically selected by the Display software represented by colored circles.

**Strategy 3.** A pair of in-phase and out-of-phase images were obtained from the central region with the best positioning of the liver. For each pair, four ROIs, measuring 1 cm^2^ each, were determined in segments VI/VII, V/VIII, IV, and II/III, equally for both groups. After the signal intensity of the four ROIs had been measured, the mean signal intensity was calculated, which was the basis for calculating the liver fat fraction (Figure 5).

In all three strategies, the ROIs were placed in order to exclude areas with large intrahepatic blood vessels or liver lesions of another nature. That precluded any inappropriate measurements.

In the statistical analysis, a paired Student-t test was used in order to compare ages between the two groups. The samples from both groups were nonparametric, as determined by the Shapiro-Wilk test. Repeated-measures analysis of variance with Bonferroni’s post-test was used in comparisons among the strategies in each group. The intraclass correlation coefficient was calculated to determine the level of interobserver agreement. Values of *p* < 0.05 were considered statistically significant.

## RESULTS

We selected 218 MRI examinations of the abdomen of patients with hepatic steatosis. Of those, 16 were excluded: nine because of the presence of movement artifacts or magnetic susceptibility artifacts; and seven because of the presence of multiple liver lesions of another nature that could not be omitted from the ROI.

Among the 202 examinations included in the study, the pattern of fatty infiltration was homogeneous in 165 (81.7%) and heterogeneous in 37 (18.3%). Therefore, the homogeneous group comprised 37 MRI examinations, matched to the 37 examinations in the heterogeneous group.

Each group consisted of 19 women and 18 men. The mean age of the patients was 53.1 ± 15.5 years in the homogeneous group and 50.3 ± 7.01 years in the heterogeneous group. As shown in Table 1, there was no significant difference between the groups regarding age (*p* = 0.31).

**Table 1:** Ages of patients with hepatic steatosis, by presentation form and patient sex.

Form	Age (years), mean ± standard deviation (range)		
	All patients	Men	Women
Homogeneous	53.1 ± 15.5 (22-77)	54.2 1 5.6 (24-78)	52.1 ± 13.4 (22-68)
Heterogeneous	50.3 ± 7.0 (19-75)	48.3 ± 13.5(19-72)	52.2 ± 112.4 (25-75)

**Figure 6:**
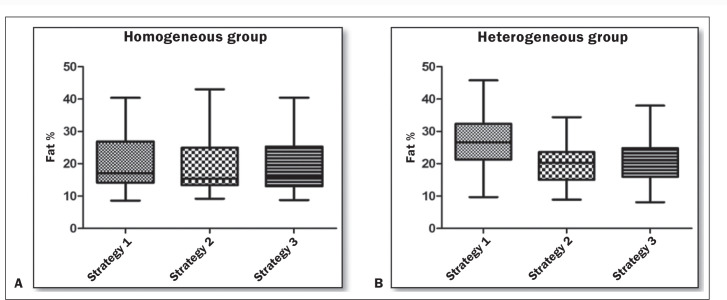
Variation in fat fraction. Graphs showing variations in the fat fraction with the three strategies, in the homogeneous group (A) and in the heterogeneous group (B).

The distribution and variation of the liver fat fraction values in the two groups, for each strategy, are illustrated in Figure 6, and the mean values are shown in Table 2. There was no statistically significant difference among the three strategies in the homogeneous group (*p* = 0.69). However, in the heterogeneous group, there was a strong statistically significant difference among the strategies (*p* < 0.0001).

**Table 2:** Hepatic fat fraction calculated by MRI, with three different strategies.

Group	Fat fraction (%), mean ± standard deviation (range)			*P*	F
	Strategy 1	Strategy 2	Strategy 3		
Homogeneous	20.8% ± 8.6 (8.6%-40.4%)	19.2% ± 8.4 (9.2%-43.0%)	19.6% ± 8.2 (8.2%-40.4%)	0.69	0.36
Heterogeneous	27.5% ± 9.7 (9.7%-45.8%)	19.7% ± 6.7 (8.9%-34.4%)	20.6% ± 6.5 (8.0%-37.9%)	< 0.0001	54.1

In the heterogeneous group (Table 3), we observed greater variation in strategy 1 than in the other strategies, the mean difference being 7.7 for strategy 1 versus strategy 2 and 6.9 for strategy 1 versus strategy 3 (*p* < 0.0001 for both). In that same group, there was no significant difference between strategy 2 and strategy 3, with a mean difference of 0.8 (*p* = 0.37). In the homogeneous group (Table 4), there were no significant differences between the strategies when compared separately.

**Table 3:** Comparison between strategies in the heterogeneous group.

Comparison	Mean difference	*P*
Strategy 1 vs. strategy 2	7.7	0.0001
Strategy 2 vs. strategy 3	0.8	0.37
Strategy 3 vs. strategy 1	6.9	0.0001

**Table 4:** Comparison between strategies in the homogeneous group.

Comparison	Mean difference	*P*
Strategy 1 vs. strategy 2	1.6	0.69
Strategy 2 vs. strategy 3	0.4	0.97
Strategy 3 vs. strategy 1	1.2	0.82

The levels of interobserver agreement on the liver fat fraction with strategies 1 and 2 are shown in Table 5. There was a strong correlation between the values found with those two strategies. There was no disagreement between the examiners, and there was no need to review the measurements obtained.

**Table 5:** Intraobserver agreement.

Strategy	Homogeneous group		Heterogeneous group	
	ICC	Cl 95%	ICC	Cl 95%
1	0.98	0.96-0.99	0.97	0.95-0.98
2	0.99	0.99-0.99	0.96	0.93-0.98

ICC, intraclass coefficient correlation; CI, confidence interval.

## DISCUSSION

Hepatic steatosis affects approximately 20% of the general population and is easily detected with conventional MRI. Because steatosis is a chronic disease with a high prevalence worldwide, is strongly associated with other comorbidities, and is potentially reversible, quantifying liver fat is important. There is a growing need, in the clinical environment and in the research field, to detect and evaluate the severity of this disease^(1,4,20,22)^. In addition, precise quantification is necessary for the longitudinal monitoring of patients^(3,22-24)^.

The most prevalent disease of the liver is MASLD, which affects approximately 25% of the population^(25-27)^. It encompasses a spectrum of diseases, including steatosis, steatohepatitis, and cirrhosis. The incidence of cancer is 1.3 times higher in patients with MASLD than in those without, the most prevalent neoplasms in such patients being hepatocellular carcinoma, gastrointestinal tumors, and breast cancer. It is estimated that 10-15% of patients with MASLD will develop cirrhosis, the risk of which is 2.5 times greater in such patients, who are also 2.0 times more likely to develop fibrosis than are those without MASLD^(26)^. Biopsy carries significant risks of complications that lead to hospitalization and death, requiring several hours of postprocedure recovery, making it unfeasible given the high prevalence of hepatic steatosis^(12,27,28)^. In addition, one of the main limitations is the lack of representation of the liver as a whole, given that it can be heterogeneous in some patients with diffuse diseases, and, consequently, the biopsy results vary widely and are highly contested. A recent study conducted by Ratziu et al.^(12)^, involving 51 patients who underwent two biopsies in close locations, demonstrated a kappa value of 0.64 for the classification of steatosis, which indicates a level of agreement that is inadequate for reliable staging. Other studies have shown significant variability in sampling when more than one sample is analyzed^(28-31)^. However, despite being an invasive method, biopsy continues to be a reference, because it allows he evaluation of not only the amount of fat in the liver but also other important histological characteristics, such as inflammation, cell damage, and the size of fat droplets^(10,12,24-28)^.

For the quantification of liver fat, MRI is a well-established method. Calculating the fat fraction by the chemical shift MRI technique is a simple and quick method. Levenson et al.^(18)^ compared the use of the Dixon method with that of semiquantitative histological evaluation by liver biopsy for the quantification of steatosis (the liver fat fraction) and reported a good correlation between them. However, to our knowledge, there have been no studies demonstrating the best way to measure signal intensity for this calculation, given that steatosis can present a heterogeneous pattern of infiltration.

In our study, we found the prevalence of the different forms of steatosis to be 18% for the heterogeneous presentation and 82% for the homogeneous presentation. In a retrospective study of abdominal computed tomography scans in a general population, El-Hassan et al.^(33)^ found the prevalence of fatty infiltration to be 9.7%, the infiltration being diffuse in 68%, focal in 9%, and multinodular in 22%. Although the heterogeneous form of hepatic steatosis is not as common as the homogeneous form, these data suggest that the former is not rare. Nevertheless, there have been few studies reporting the prevalence of the heterogeneous form.

When evaluating the quantification of steatosis by MRI separately in patients with homogeneous or heterogeneous steatosis, we observed that in the group with heterogeneous steatosis, the use of a 1 cm^2^ ROI to obtain the fat fraction in the most altered area showed significant variation, demonstrating that the evaluation of heterogeneous steatosis when performed with a small-diameter ROI, like a biopsy sample, might not be representative. However, the use of the mean of four 1 cm^2^ ROIs or segmenting a representative area to measure signal intensity did not show significant variations. Therefore, we believe that measurement strategies that use more than one ROI or the segmentation of a representative area to obtain the fat fraction in heterogeneous steatosis can provide data that are closer to reality, sometimes even more accurate than biopsy.

None of the strategies employed in the present study demonstrated a significant difference in the final value of the fat fraction in patients with homogeneous steatosis. That was an expected finding, given that the deposition of fat in the parenchyma occurs uniformly, demonstrating that there should be no concern in obtaining the fat fraction in this group of patients. In both groups, we also observed high reproducibility of the strategies that were carried out manually.

Despite its simplicity and ease of application, the chemical shift method has major limitations. In addition to the cost and limited availability of MRI examinations, the processing of images in an out-of-phase sequence using this technique results in signal intensities that represent a mixture of water and fat, making it impossible to determine which element is dominant in the image. Therefore, the method becomes less reliable for evaluating patients with severe steatosis. More recent techniques, such as measuring the fat fraction by proton density, have greater accuracy due to multiple corrections in the process of obtaining the signal, although they need to be purchased as a separate software package, making the examination even more expensive^(20,21)^. In any case, we believe that the results obtained in the present study regarding measurement strategies can also be applied to this technique. It is interesting to highlight that the strategy applied in the MRQuantif software^(21)^ is very similar to strategy 3 of the present study, with three ROIs being obtained in the liver parenchyma, rather than four as used in our study.

Limitations of our study include the retrospective nature and the small size of the sample of heterogeneous steatosis scans. The lack of a comparison of results with histological evaluation via biopsy was also a limitation of the study, although the strategies were compared with each other for the same patient.

## CONCLUSION

Quantifying liver fat is important not only for diagnosis but also to determine the severity of steatosis, to actively monitor patients, and to evaluate treatment responses. Quantification of liver fat by MRI using only one ROI has low representativeness, especially in cases of heterogeneous steatosis. There seems to be no significant difference between obtaining the mean of four ROIs and segmenting a representative area of the parenchyma.
